# Effect of Different Specimen Preparation Methods on Dentin Shear Bond Strength: Polyethylene Tubes vs Jig System

**DOI:** 10.3290/j.jad.c_2707

**Published:** 2026-06-04

**Authors:** Sena Balaban Karakaş, Mert Karakaş, Asude Mürüvet Oğuz, Hacer Deniz Arısu

**Affiliations:** a Sena Balaban Karakaş Research Assistant, Gazi University Faculty of Dentistry, Department of Restorative Dentistry, Biskek St. 1, St. Number 8, Cankaya, Ankara, Turkey. Study concept and design, material preparation, data collection and analysis, drafted the initial version of the manuscript, critically revised and approved the final version.; b Mert Karakaş Research Assistant, Gazi University Faculty of Dentistry, Department of Restorative Dentistry, Biskek St. 1, St. Number 8, Cankaya, Ankara, Turkey. Study concept and design, material preparation, data collection and analysis, drafted the initial version of the manuscript, critically revised and approved the final version.; c Asude Mürüvet Oğuz Research Assistant, Gazi University Faculty of Dentistry, Department of Restorative Dentistry, Biskek St. 1, St. Number 8, Cankaya, Ankara, Turkey. Study concept and design, material preparation, data collection and analysis, drafted the initial version of the manuscript, critically revised and approved the final version.; d Hacer Deniz Arısu Professor, Gazi University Faculty of Dentistry, Department of Restorative Dentistry, Biskek St. 1, St. Number 8, Cankaya, Ankara, Turkey. Study concept and design, material preparation, data collection and analysis, critically revised and approved the final version.

**Keywords:** dentin, dental bonding, shear strength

## Abstract

**Purpose:**

This study evaluated the influence of different specimen preparation methods on the shear bond strength (SBS) of a universal adhesive to dentin, with the aim of identifying a more standardized and reliable protocol for SBS testing.

**Methods and Materials:**

Thirty-three extracted molars were used to obtain 66 dentin surfaces. After surface preparation with 600-grit silicon carbide paper, specimens were randomly assigned to three groups (n = 22) according to the specimen preparation technique: tube-removed (polyethylene tubes removed before testing), tube-retained (tubes left in place during testing), and jig-based (Ultradent jig system, South Jordan, UT, USA). SBS testing was performed using a universal testing machine at 1 mm/min. Failure modes were analyzed under a stereomicroscope and representative specimens were examined by scanning electron microscopy (SEM). Data were analyzed using one-way ANOVA and Tamhane’s T2 post-hoc test (α = 0.05).

**Results:**

Significant differences were found among the groups (*P* < 0.001). The highest mean SBS values were obtained in the jig-based group, followed by the tube-removed and tube-retained groups. Adhesive failure was predominant across all groups, with 100% adhesive failures observed in the jig-based group. SEM analysis revealed a more clearly delineated bonding interface in the jig-based group, while tube-based groups exhibited minor polyethylene residues.

**Conclusion:**

These findings indicate that the specimen preparation method plays a critical role in the reliability and interpretation of SBS testing outcomes. The jig-based approach may provide more consistent and reproducible SBS results compared to tube-based methods, highlighting the importance of standardized specimen fabrication in adhesive dentistry research.

The performance and durability of contemporary adhesive restorations depend largely on the stability of the adhesive interface, which must withstand long-term mechanical, thermal, and chemical stresses in the oral environment.^23^ Although clinical studies provide the most reliable evidence for evaluating the long-term success of adhesive systems, they are time-consuming, costly, and influenced by multiple confounding variables that make it difficult to isolate the adhesive interface as the sole determinant of restoration failure. Therefore, *in vitro* tests remain an indispensable and efficient approach for obtaining standardized and reproducible data under controlled experimental conditions.^15,24,27^

Among laboratory methods, the shear bond strength (SBS) test is widely used due to its relatively simple protocol and the absence of specimen sectioning. Moreover, SBS testing allows failure mode analysis, which provides insight into the integrity of the adhesive interface.^26,27^ However, SBS outcomes are highly sensitive to experimental variables, including substrate type, loading configuration, and specimen preparation methods. Specimen preparation is often treated as a minor procedural detail rather than a critical methodological variable, potentially compromising inter-study comparability. Such methodological variability can lead to substantial discrepancies among studies and complicate the interpretation and clinical relevance of reported bond strength values.^1,3,24^ Therefore, improving the methodological standardization of SBS testing remains essential for ensuring meaningful interpretation and inter-study consistency.

One key source of methodological variability in SBS testing is the approach used to confine and shape the composite resin during specimen preparation. Several mold systems, including metal, polyethylene, one- or two-piece designs, have been proposed to standardize the bonded area.^5,6,8,26^ However, differences in mold rigidity and handling properties may influence interfacial stress distribution and potentially introduce microdefects during specimen fabrication.^9,20,26^ Polyethylene tubes are commonly used in conventional SBS protocols, yet studies differ on whether these tubes should be removed before testing.^14,19^ or retained during loading.^10,13,28^ Only a limited number of investigations have directly compared these approaches, and findings remain inconsistent.^2,3^ More recently, commercially manufactured jig systems (Ultradent, South Jordan, UT, USA) have been developed to improve specimen alignment and geometric standardization^16,18,25,27^; however, their impact on measured bond strength values has not been extensively explored. Therefore, further clarification of how specimen preparation protocols influence SBS outcomes is needed.

This study aimed to compare three specimen preparation methods for the SBS test: (1) polyethylene tubes removed before testing; (2) polyethylene tubes retained during testing; and (3) commercially available jig systems. The null hypothesis tested was that the specimen preparation methods would not significantly affect the dentin bond strength values.

## METHODS AND MATERIALS

### Power Analysis

The total sample size was determined to be 66 (22 specimens per group) using the G-Power software package (G-Power Ver. 3.0, Germany),^11,12^ with a Type I error rate (α) of 0.05, statistical power (1 – β) of 0.80, and an effect size (f) of 0.40 as suggested by Cohen.^17^

### Materials

The materials used in this study, along with their compositions and manufacturers’ instructions, are presented in Table 1.

**Table 1:** Materials used in the study and their compositions

Manufacturer	Material	Chemical compositions
Scotchbond Universal Plus Adhesive, 3M ESPE, St. Paul, MN, USA	Universal adhesive	MDP*, HEMA*, Vitrebond copolymer, dimethacrylate resins, ethanol, water, initiators, dual-cure accelerator, optimized mixture of silane, filler
Filtek Z550, 3M ESPE, St. Paul, MN, USA	Nanohybrid composite resin	Bis-GMA*, UDMA*, Bis-EMA*, PEGMA*, TEGMA*, 78.5% filler
*Abbreviations: MDP: Methacryloyloxydecyl dihydrogen phosphate, HEMA: 2-Hydroxyethylmethacrylate, Bis-GMA: Bisphenol-A-glycidyl methacrylate, UDMA: Urethane dimethacrylate, Bis-EMA: Bisphenol-A-ethoxylated-glycidyl dimethacrylate, PEGMA: Polyethylene glycol monomethacrylate, TEGMA: Triethylene glycol dimethacrylate.		

### Specimen Preparation

Thirty-three extracted, non-carious, crack-free human molars indicated for extraction were included in this study. The teeth used in this study were collected from the outpatient clinic of the Department of Oral and Maxillofacial Surgery, after obtaining patient consent for the use of extracted impacted third molars for research purposes. The use of extracted human teeth was approved by the Ethics Board of the Gazi University Faculty of Dentistry (approval number: GUDHKAEK.2023.07/9). All procedures were performed in accordance with the Declaration of Helsinki and its later amendments. The crowns were sectioned from the roots using a low-speed diamond saw under water-cooling, and each crown was then sectioned mesiodistally to obtain 66 dentin specimens. The specimens were embedded in self-cured acrylic resin within polyvinyl chloride cylinders, leaving the dentin surface exposed. Each surface was polished with 600-grit silicon carbide paper under water-cooling for 60 s to obtain a flat, standardized smear layer. All specimen preparation procedures were performed following a standardized workflow by a single operator to minimize operator-dependent variability.

### Experimental Groups

The specimens were randomly assigned to three groups (n = 22) according to the specimen preparation method. In the tube-based groups, a polyethylene tube was positioned on the dentin surface prior to adhesive application to delimit the bonding area. In the jig-based group, the bonding area was delimited by the jig system. A universal adhesive (Scotchbond Universal Plus, 3M ESPE, St. Paul, MN, USA) was applied to the dentin surfaces following the manufacturer’s instructions and light-cured for 10 s. A nanohybrid composite resin (Filtek Z550, 3M ESPE) was then placed according to the procedures assigned to each group to obtain a standardized loading area with a diameter of 2.4 mm and a height of 3 mm. The composite was applied in two increments, and each layer was light-cured separately for 20 s. All adhesive and composite polymerizations were performed using an LED light-curing unit (Valo X, Ultradent, South Jordan, UT, USA) operating at 1350 mW/cm^2^ and connected to a power supply to prevent fluctuations in light intensity.

**Fig 1a and b:**
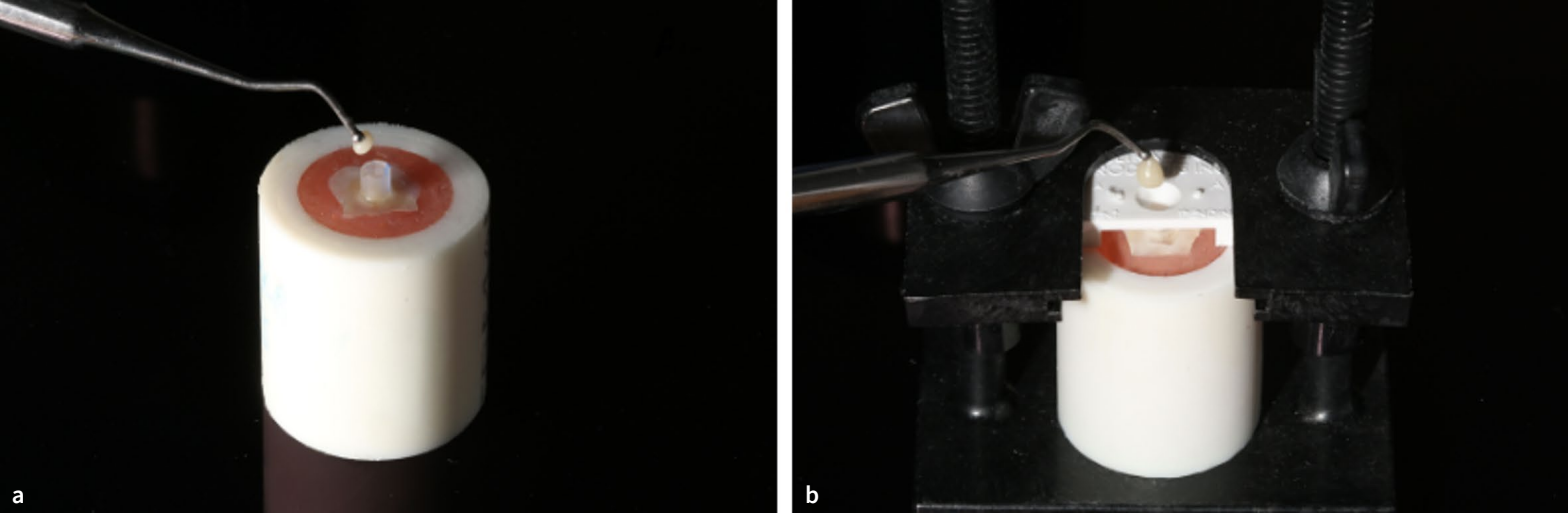
Specimen preparation method using polyethylene tubes (a) and a commercial jig system (b). (a) Composite resin placement with a polyethylene tube positioned on the dentin surface (used in tube-based groups). (b) Composite resin placement with a jig system (Ultradent, South Jordan, UT, USA) (used in jig-based group).

Tube-removed group: A polyethylene tube with an inner diameter of 2.4 mm (outer diameter: 2.9 mm) was positioned on the dentin surface to form a standardized composite cylinder, and the composite resin was incrementally placed inside the tube (Fig 1a). The specimens were then stored in distilled water at 37°C for 24 h, after which the tubes were carefully removed using a scalpel.
Tube-retained group: The same bonding and composite placement protocol was used (Fig 1a); however, the polyethylene tubes with an outer diameter of 2.4 mm (inner diameter: 1.9 mm) were left in place during SBS testing following 24 h of water storage. In this group, the tube-composite complex was considered part of the loading configuration during shear force application. Accordingly, the experimental design was configured such that the combined external diameter of the polyethylene tube and the enclosed composite cylinder was standardized to 2.4 mm, in order to equalize the effective loading area across all groups.
Jig-based group: Composite cylinders were fabricated using a commercial bonding jig (Ultradent jig system, Ultradent, South Jordan, UT, USA) (Fig 1b). Following light curing, the jig was removed, and the specimens were stored in distilled water at 37°C for 24 h prior to testing.

### Shear Bond Strength Test

**Fig 2a to c:**
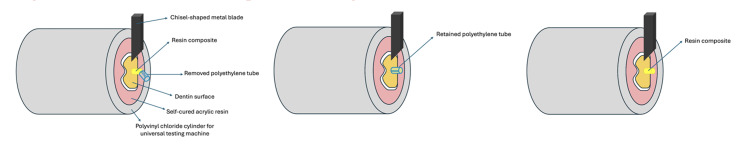
Schematic illustration of specimen configurations and shear force direction. (a) Tube-removed group; (b) Tube-retained group; (c) jig-based group.

SBS was measured using a universal testing machine (Shimadzu AG-IS, Kyoto, Japan) at a crosshead speed of 1 mm/min. A chisel-shaped metal blade was aligned parallel to the adhesive interface and loaded until failure occurred. The maximum load (N) was recorded. For shear bond strength calculation, the diameter of the area subjected to shear loading was measured individually for each specimen using a digital caliper prior to testing. The recorded load values were converted to bond strength (MPa) by dividing the maximum load by the actual measured cross-sectional area of each specimen (πr^2^). A schematic illustration of the three specimen configurations and the direction of the applied shear force is presented in Figure 2.

### Failure Mode Analysis

After testing, all fractured specimens were examined under a stereomicroscope (Olympus, Tokyo, Japan) at ×40 magnification to determine the failure mode. Failures were categorized as:

Adhesive failure: failure at the adhesive interface,
Cohesive failure: failure within the composite resin or dentin,
Mixed failure: simultaneous occurrence of adhesive and cohesive failures.^22^

Representative specimens from each group were sputter-coated with gold and examined using scanning electron microscopy (SEM, Hitachi, Japan) at ×45 magnification. SEM micrographs were acquired at low magnification to provide an overall morphological overview of the fractured specimens.

### Statistical Analysis

Data were analyzed using IBM SPSS Statistics 25.0 (IBM, Armonk, NY, USA). According to the Shapiro–Wilk test results, the data were normally distributed across the groups; however, as Levene’s test indicated that the assumption of homogeneity of variances was not met, the ANOVA F-test and Tamhane’s T2 post-hoc tests were employed for statistical comparisons. The significance level was set at α = 0.05.

## RESULTS

### Shear Bond Strength Test Results

The SBS test results are presented in Table 2. Statistically significant differences were observed among the groups (*P* < 0.05). The highest mean SBS value was obtained in the jig-based group, followed by the tube-removed group and the tube-retained group, which exhibited the lowest values.

**Table 2:** Shear bond strength values of groups

Groups	N	Shear bond strength values (Mpa)	
		Mean	Std. deviation
Tube-removed	22	16.90b	5.88
Tube-retained	22	8.72a	3.83
Jig-based	22	23.04c	7.97
ANOVA (*P* = )		< 0.001	
F		30.213	
η^2^		0.490	
Different lower-case letters in the same column indicate the difference in shear bond strength between groups according to the Tamhane T2 post hoc test.			

### Failure Mode Analysis Results

**Fig 3:**
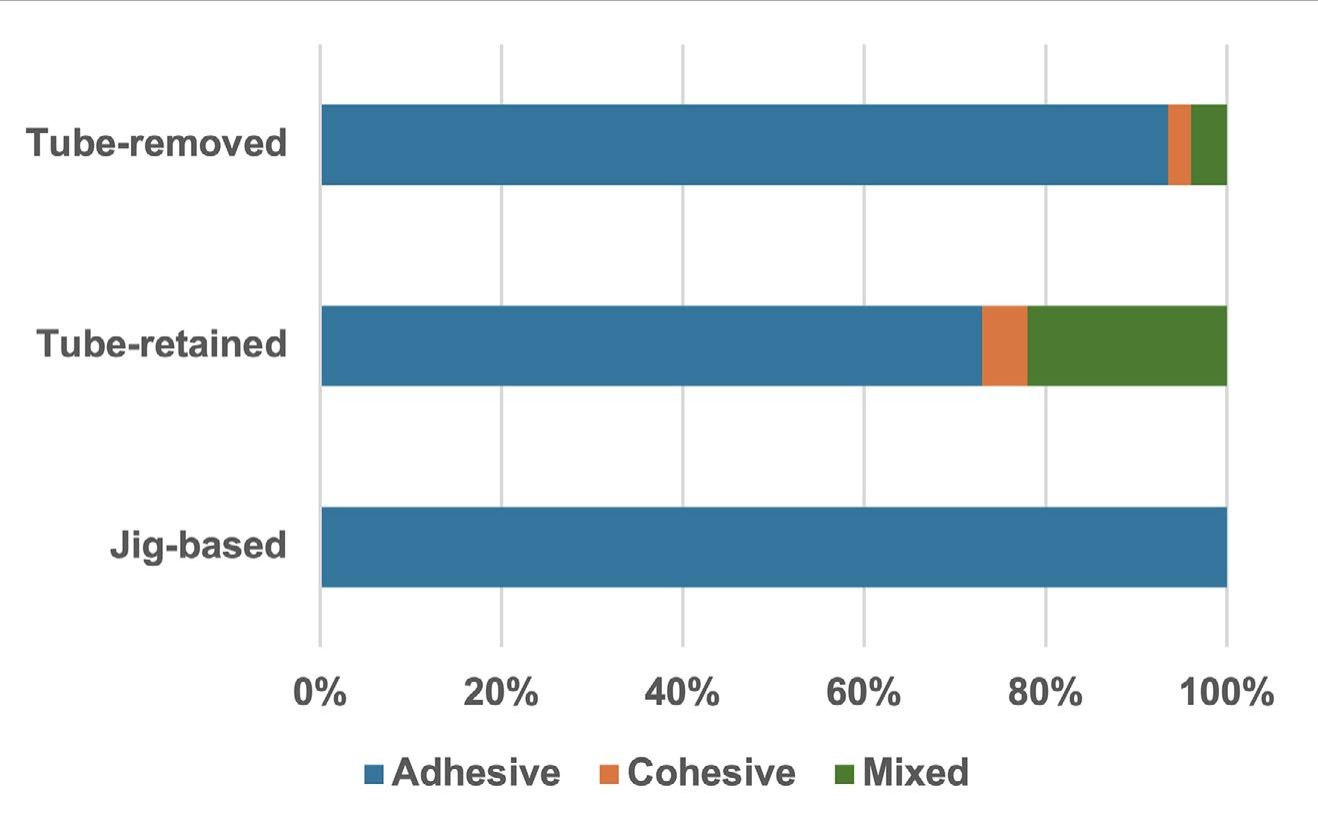
Distribution of failure modes (adhesive, cohesive, mixed) among the test groups.

The distribution of failure modes is shown in Figure 3. Adhesive failure was predominant across all experimental groups. In the jig-based group, all specimens (100%) exhibited adhesive failure, while the tube-removed and tube-retained groups showed a combination of adhesive, cohesive, and mixed failure patterns.

### SEM Analysis

**Fig 4:**
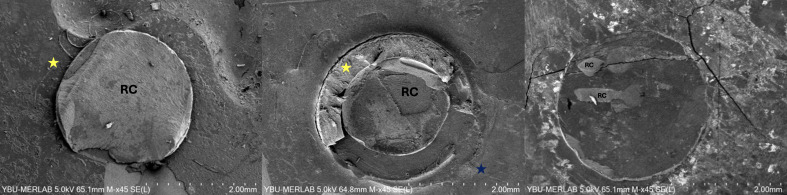
SEM micrographs of the fractured surfaces. RC = composite resin; yellow star = polyethylene tube fragments; blue star = bonded interface extended beyond the tube-defined boundary.

SEM micrographs of the fractured surfaces are presented in Figure 4:

**Image A**, corresponding to the tube-removed group, shows a cohesive failure within the composite mass. The yellow star indicates residual polyethylene tube fragments adhering to the dentin surface. Although adhesive and mixed failures predominated in this group, this image shows a less frequently observed (exceptional) cohesive failure.
**Image B**, obtained from the tube-retained group, displays a mixed failure mode, where both adhesive and cohesive fractures coexist. The yellow star denotes indistinct polyethylene tube fragments, whereas the blue star highlights an area where the bonded interface extended beyond the tube-defined boundary. In this group, the combined diameter of the polyethylene tube and composite was standardized to 2.4 mm.
**Image C**, obtained from the jig-based group, exhibits a predominantly adhesive failure pattern characterized by a clean dentin surface and minimal remnants of composite resin (RC).

## DISCUSSION

Because the resin–dentin interface is the most critical and vulnerable component of adhesive restorations, reliable laboratory assessment of this interface is essential for interpreting the performance of adhesive systems. Despite its well-recognized limitations, the SBS test remains one of the most frequently used methods in adhesive dentistry research due to its procedural simplicity, the absence of sectioning requirements, and its relatively low incidence of pre-test failures. In addition, evaluation of failure modes provides complementary insight by helping to distinguish whether the measured values reflect true interfacial adhesion or are influenced by cohesive properties of the substrate or the restorative material. However, the validity and reproducibility of SBS testing depend heavily on the standardization of specimen geometry, bonding area, and loading configuration.^2,15^

The present study demonstrated statistically significant differences in dentin SBS values among the different specimen preparation methods. Accordingly, the null hypothesis stating that the specimen preparation method would not influence SBS outcomes was rejected. The present findings demonstrate that the specimen preparation method is a determining factor that should be explicitly considered and reported in SBS testing. In light of these findings, improving the methodological standardization of specimen preparation in SBS testing may enhance its scientific value and inter-study comparability.

Previous studies have examined the effect of mold configuration on bond strength test outcomes, although reported results remain inconsistent—likely due to variations in mold rigidity and loading configurations. Ayar et al reported that the type of mold, whether silicone, one-piece plexiglass, or two-piece removable plexiglass, had no significant influence on the measured bond strength.^5^ In contrast, Cheetnam et al suggested that the use of rigid metallic molds may reduce bending moments during loading, resulting in higher bond strengths.^8^ Similarly, Barkmeier et al found that specimens confined within metallic molds exhibited greater bond strength than unsupported specimens.^6^ Notably, these studies exclusively employed rigid molds. In contrast, the present study employed flexible polyethylene tubes, which are commonly used in SBS testing protocols. Rigid molds may promote more direct stress transmission to the adhesive interface, whereas flexible molds such as polyethylene tubes may partially dissipate applied forces, resulting in more complex stress distributions. These methodological differences highlight that variations in specimen configuration and loading conditions may substantially influence SBS outcomes and should be carefully considered when comparing results across different studies.

Given the widespread use of polyethylene tubes in SBS testing, differences in specimen preparation methods, particularly whether the tubes are retained or removed prior to loading, warrant careful consideration. While some researchers have performed SBS testing with the tubes retained during loading,^10,13,28^ others have removed the tubes prior to testing using a scalpel.^14,19^ However, this methodological aspect has received limited attention in the literature, particularly with respect to direct comparisons among different specimen preparation methods.^2,3^ In addition to these two commonly used approaches, the present study incorporated a prefabricated jig system, enabling direct comparison of tube-retained, tube-removed, and jig-based specimen preparation methods. Because the presence or absence of a polyethylene tube during shear loading may alter the path of force transmission to the adhesive interface, such methodological differences may contribute to variability in reported bond strength values across studies.

The highest mean SBS values were obtained in the group prepared using the Ultradent jig system, followed by the tube-removed group, whereas the lowest values were recorded when the tubes were retained during testing. The lower bond strength observed in the tube-retained group may be associated with the elastic nature of polyethylene tubes, which may partially absorb applied shear forces rather than transmitting them directly to the adhesive interface, potentially resulting in non-uniform stress distribution and earlier interfacial failure.^2^ Such differences in force transmission and stress distribution may partly explain the inconsistent SBS outcomes reported among studies employing different specimen preparation methods.

In contrast to the present findings, Andrade et al^2^ reported that removal of polyethylene tubes prior to testing did not significantly influence the resulting bond strength, although the authors noted that the mechanical stress generated during tube cutting could induce microcracks or interfacial strain, potentially contributing to premature failures. However, an important methodological difference should be considered. In their study, the composite resin was polymerized prior to bonding to the dentin, which does not fully replicate clinical bonding procedures.^2^ Polymerization shrinkage stresses are known to influence the adhesive interface. In the present study, the composite was light-cured directly on the dentin surface in all groups, a protocol that more closely reflects clinical conditions and may partially explain the differences observed between studies. In addition to differences in polymerization sequence, variations in specimen configuration and loading conditions may have further contributed to the discrepancies between the findings of Andrade et al^2^ and the present study.

The higher bond strength observed in the jig-based group may be associated with the rigid and standardized configuration of the jig, which provides a stable bonding geometry and may promote a more homogeneous distribution of stresses during loading. Previous reports have suggested that test setups yielding comparatively higher bond strength values may also demonstrate greater sensitivity in discriminating subtle differences among materials and application variables.^3,8,24^ Such improved standardization of specimen geometry and stress transfer may enhance the consistency of *in vitro* bond strength measurements and thereby support a more clinically meaningful interpretation of laboratory findings.

Several authors have noted practical limitations associated with the use of polyethylene tubes in SBS testing.^20,26^ Tedesco et al and Pires et al reported that inserting the composite into these tubes may trap air bubbles and create peripheral gaps, while inadequate adaptation of the tubing to the dentin surface may lead to variations in specimen geometry.^26^ Foong et al further hypothesized that polyethylene tubing may alter the oxygen-inhibited layer or influence adhesive layer thickness, potentially affecting the interfacial bond.^13^ In the present study, similar handling difficulties were occasionally encountered, particularly when the tubes detached from the dentin surface during composite placement. Although such specimens were re-prepared and not categorized as pre-test failures, these operator-dependent variables may limit methodological standardization. Moreover, the presence of residual polyethylene fragments observed in the SEM images suggests that a portion of the recorded bond strength may be influenced by tube-dentin interaction rather than solely reflecting the intrinsic strength of the adhesive interface.

Tedesco et al reported that stresses generated during the manual removal of polyethylene tubes could lead to premature fractures and lower measured bond strength.^26^ However, in the present study, specimens in which the tubes were removed prior to testing demonstrated higher bond strength values compared to those in which the tubes were retained. This suggests that, under the experimental conditions of the present study, tube removal did not appear to introduce substantial additional stress. Nonetheless, the removal of elastic molds remains a procedural step in which localized mechanical stress could occur, depending on the operator’s technique. Therefore, these findings underscore the need to reduce operator-dependent variation and to employ specimen preparation methods that enhance standardization and consistency in SBS testing.

In the present study, adhesive failures predominated in the jig-based group. Although a high frequency of adhesive failures has been interpreted in the literature as an indication that applied stresses are effectively transmitted to the adhesive interface, thereby supporting the validity of the test and the assessment of adhesive performance, failure mode distribution alone does not provide definitive information regarding stress transmission.^4,8,9^ Accordingly, failure mode distribution should be interpreted with caution and in conjunction with other methodological considerations.^7,21^

Nevertheless, within the context of the present study, the consistent occurrence of adhesive failures in the jig-based group may be regarded as a methodological advantage, suggesting that the testing configuration facilitated stress concentration at the adhesive interface rather than within the surrounding substrates. This outcome may be associated with the rigid and standardized configuration of the jig, which promotes preferential stress transfer to the adhesive interface during shear loading. As a result, stress concentration is more likely to occur at the resin–dentin interface, leading predominantly to adhesive failure. Moreover, SEM micrographs from this group revealed a well-defined and continuous bonding interface, consistent with the higher bond strength values observed. In contrast, when polyethylene tube is retained during testing, its elastic nature may allow partial dissipation of the applied shear force and redistribution of stresses within the surrounding composite and dentin. Such heterogeneous stress fields may increase the likelihood of mixed or cohesive failures. Taken together, these findings suggest that the jig-based specimen preparation may help reduce operator-dependent variability and enhance the reliability and discriminative capacity of SBS testing.

The findings of this study should be interpreted in consideration of its *in vitro* design and methodological limitations. Although efforts were made to standardize all experimental steps, manual procedures, particularly during the placement and removal of polyethylene tubes, may have introduced some operator-related variability. In addition, stress distribution during loading was not directly evaluated; therefore, future studies incorporating finite element analysis or digital image correlation may help provide complementary insight into stress transmission associated with different specimen preparation methods in SBS testing. Another limitation is that only one universal adhesive and one nanohybrid composite were evaluated. Since the behavior of adhesive interfaces may vary with adhesive composition, filler content, and elastic modulus of composite resins, comparing filled versus unfilled adhesives and composite resins with different elastic moduli may broaden understanding of how material characteristics interact with specimen preparation. Furthermore, the present findings are limited to a universal adhesive-composite system and cannot be directly extrapolated to other bonding strategies, such as self-adhesive resin cements. Given the differences in chemical composition, viscosity, and bonding mechanisms among adhesive materials, stress transmission and interfacial behavior may differ under similar testing conditions.

## CONCLUSION

Within the limitations of this *in vitro* study, the specimen preparation method significantly influenced the dentin SBS outcomes. Specimens prepared using the Ultradent jig system demonstrated higher and more consistent SBS values compared to tube-based methods. These findings suggest that jig-based specimen preparation may enhance the consistency and reliability of SBS testing by providing a more standardized bonding geometry and reducing operator-dependent variability. Therefore, standardization of specimen preparation procedures is essential for improving the reproducibility and comparability of bond strength data in adhesive dentistry studies.

### Acknowledgments

#### Clinical relevance

Standardized specimen preparation methods may enhance the reproducibility and comparability of SBS results, thereby improving the clinical interpretability of laboratory data on adhesive performance. Although *in vitro* outcomes cannot be directly extrapolated to clinical outcomes, greater methodological standardization may facilitate more meaningful comparisons among adhesive systems and assist clinicians in making evidence-based material selections. In this context, the use of a jig-based specimen preparation method may contribute to improved methodological standardization and stress control, which may facilitate a more reliable clinical interpretation of *in vitro* bond strength data.

#### Conflict of interest

The authors declare that there are no conflicts of interest related to the authorship or publication of this manuscript.

#### Funding

No funding was received for this study.

#### Ethical approval

This study was approved by the Ethics Board of the Gazi University Faculty of Dentistry (GUDHKAEK.2023.07/9).
